# Palliative care in advanced Huntington’s disease: a scoping review

**DOI:** 10.1186/s12904-023-01171-y

**Published:** 2023-05-03

**Authors:** Dorine J. Boersema-Wijma, Erik van Duijn, Anne-Wil Heemskerk, Jenny T. van der Steen, Wilco P. Achterberg

**Affiliations:** 1 Present Address: Department of Public Health and Primary care, Leiden University Medical Center, Hippocratespad 21, 2333 ZD Leiden, the Netherlands; 2 Present Address: Huntington Center of Expertise Topaz Overduin, Nachtegaallaan 5, 2225 SX Katwijk, the Netherlands; 3 Radboudumc Alzheimer center and Department of Primary and Community Care, Radboud university medical center, Geert Grooteplein Noord 21, 6500 HB Nijmegen, the Netherlands

**Keywords:** Huntington’s Disease, Palliative Care, Advance Care Planning, Hospice Care, Terminal Care, Neurodegenerative diseases

## Abstract

**Background:**

As Huntington’s disease (HD) is a progressive disease for which there is no cure yet, patients in the advanced stage of HD may benefit from palliative care.

**Objective:**

To review the literature focusing on palliative care in advanced stage HD, and the level of evidence.

**Methods:**

Publications between 1993 and October 29th, 2021 from 8 databases (Embase, Web of Science, Cochrane, Emcare, PsycINFO, Academic Search Premier, PMC PubMed Central and Pubmed) were included. The literature was deductively classified based on topics that are part of the definition of palliative care, or as care-related topics that emerged from the literature. Levels of evidence I (high) – V (low) were determined as defined by the Joanna Briggs Institute.

**Results:**

Our search resulted in 333 articles, 38 of which were included. The literature covered four domains of palliative care: physical care, psychological care, spiritual care, and social care. Four other topics in the literature were: advance care planning, end-of-life needs assessments, pediatric HD care, and need for health care services. Most literature was underpinned by a low level of evidence, except for the topics on social care (Level III-V), advance care planning (Level II-V) and end-of-life needs assessments (Level II-III).

**Conclusions:**

To deliver adequate palliative care in advanced HD, both general and HD-specific symptoms and problems need to be addressed. As the level of evidence in existing literature is low, further research is essential to improve palliative care and to meet patient’s wishes and needs.

**Supplementary Information:**

The online version contains supplementary material available at 10.1186/s12904-023-01171-y.

## Introduction

Huntington’s disease (HD) is a hereditary neurodegenerative disorder with an autosomal dominant mode of inheritance. It is characterized by movement disorders such as chorea, neuropsychiatric symptoms and progressive neurocognitive impairments [2]. Additionally, patients may experience symptoms such as impaired communication, swallowing problems, and obstipation [2]. The first clinical manifestations usually occur in the third or fourth decade of life [3], and the average life expectancy is about 15 to 20 years after diagnosis [4]. Death often results from aspiration pneumonia, cardiovascular diseases, cachexia [5–10], or suicide, which is more prevalent than in the general population [11, 12]. To date there is no effective treatment for the disease.

Patients become increasingly dependent on caregivers as the disease progresses and daily functioning is often impaired to the extent that patients are admitted to health care services for specialized care [9, 10, 13]. In the advanced stage of HD, optimizing quality of life (QoL) may include management of pain and other symptoms, personalized social activities and psychological and spiritual support.

The commonly used World Health Organization’s (WHO) definition of palliative care states that it is “an approach that improves the quality of life of patients and their families who are facing problems associated with life-threatening illness. It prevents and relieves suffering through the early identification, correct assessment and treatment of pain and other problems, whether physical, psychosocial or spiritual” [14]. According to the WHO, multidimensional definition, multidisciplinary treatment and personalized approaches are required, that can be provided by both professionals and caregivers, such as relatives, neighbors and friends, and by volunteers [15].

Palliative care can start long before the expected time of death, by integrating it ‘upstream’, namely by recognizing the needs of patients and family earlier on and throughout the course of the disease [16]. Palliative care has its origins in cancer care, but is important for all kinds of chronic and progressive diseases, including HD [17]. Its relevance in addressing needs may increase with increasing severity and progression of the disease, with increasing loss of functional capacity. In this scoping review we analyzed published literature on palliative care for persons in the advanced stage of HD.

## Methods

On October 29, 2021 we searched the literature using the following MesH terms: ‘Huntington’s Disease’ *and* (‘Palliative care’ *or* ‘Hospice and Palliative Care Nursing’ *or* ‘Palliative Medicine’ *or* ‘Terminal Care’) in eight databases: Embase, Web of Science, Cochrane, Emcare, PsycINFO, Academic Search Premier, PMC PubMed Central and Pubmed (for full search strategies, see Supplementary Table 1). Articles were included if they met the following inclusion criteria: English language, published after 1993 (year of identification of the causative gene expansion), focusing on aspects of palliative care, and advanced stage of HD. Synonyms that were used to include patients in the advanced disease stage were ‘advanced’, ‘late’ or ‘terminal stage’, or ‘Total Functional Capacity (TFC) score 0–6’[18]. We excluded articles on pharmacological management of physical and psychiatric symptoms, because this is not specific to the palliative phase.

First, title and abstract were screened by DJB-W, and excluded if they did not meet the inclusion criteria. Second, full-text articles were screened. In case of doubt, two other authors (EvD and A-WH) discussed whether the article fulfilled the inclusion criteria. For this scoping review, we used the ‘Preferred Reporting Items for Systematic reviews and Meta-Analyses extension for Scoping Reviews’ [19]. Data abstracted from the articles were year of publication, country, design, number of participants and topics described. Levels of evidence were assessed using the Joanna Briggs Institute Levels of Evidence 2013 [20].

## Results

**Fig. 1:**
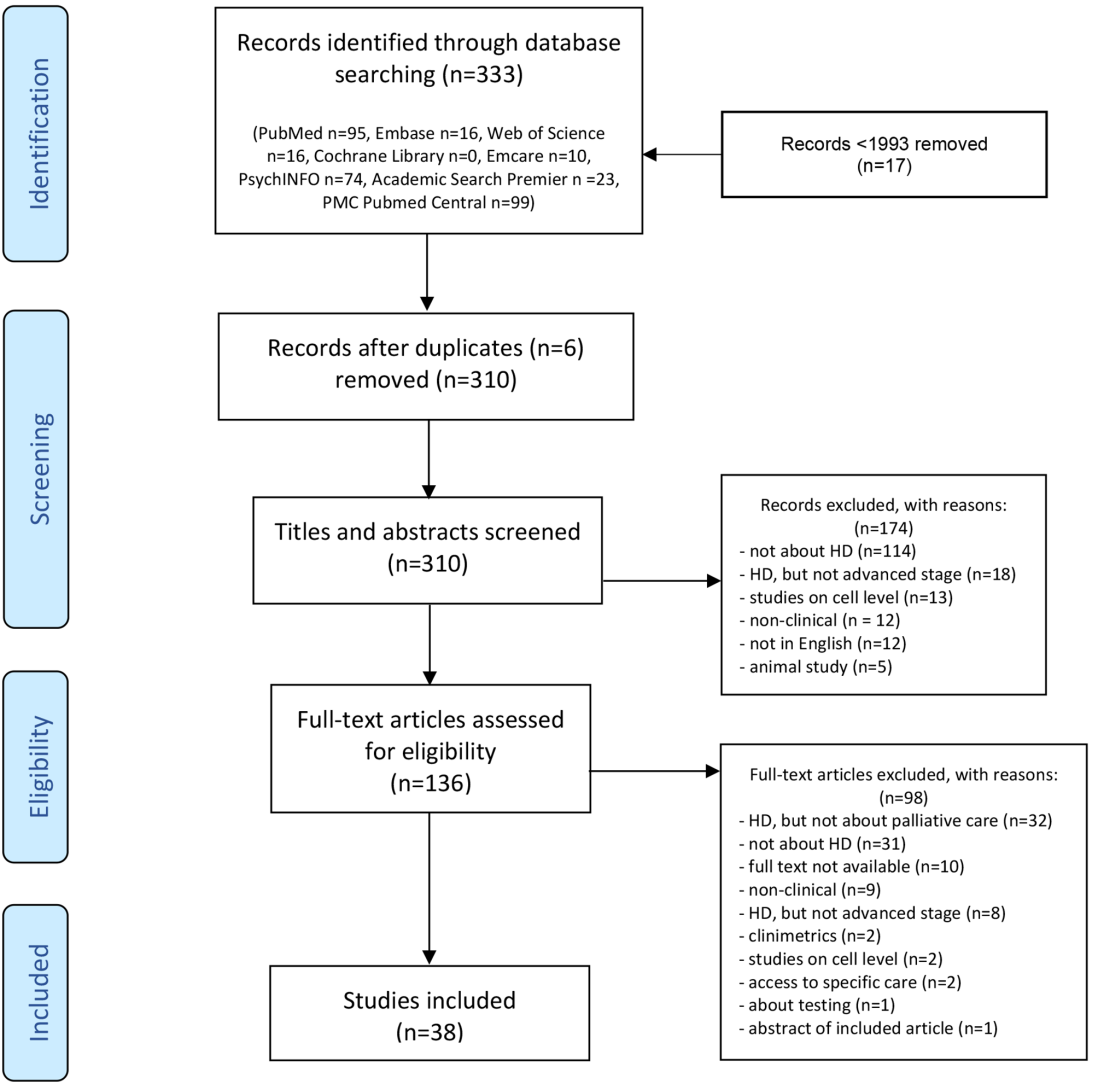
Flowchart of the search and selection process

Figure 1 shows that 310 unique records were found, of which 136 articles were evaluated as full-text. Thirty-eight articles met the inclusion criteria. The literature was classified deductively based on four topics that are part of the definition of palliative care: physical care, psychological care, spiritual care, and social care [14]. Additionally, we inductively identified four topics (advance care planning, end-of-life needs assessments, pediatric HD care, and health care services) as they were presented as important or frequently emerged from the literature, and did not fit well within the predefined four topics deduced from the palliative care definition.

Tables 1 and 2 show included articles and their levels of evidence: three surveys with evidence Level II, 13 surveys with evidence Level III, one case study and four cross-sectional studies with evidence Level IV, and 12 narrative reviews, one guideline, and four case reports with evidence Level V.

**Table 1:** Descriptive information on the included articles, in alphabetical order

No.	Author(s)	Publication date Country	Design/ Level of evidence	Participants	Topic
1	Aubeeluck et al.	2011 UK	Narrative review V	n/a	Physical care Psychological care Social care Advance care planning Health care services
2	Booij et al.	2013 the Netherlands	Survey III	14 HD patients, 5 going to daycare facility or living in a nursing home	Advance care planning
3	Booij et al.	2013 the Netherlands	Survey III	15 physicians (4 general practitioners, 7 nursing home physician, 2 psychiatrists, 2 neurologists)	Advance care planning
4	Booij et al.	2014 the Netherlands	Survey III	134 HD patients visiting out-patient clinics; mean TFC 8.5 (3.9)	Advance care planning
5	Brotherton et al.	2012 UK	Guideline V	n/a	Physical care
6	Carlozzi et al.	2016 USA	Survey II	507 manifest or pre-manifest HD patients (117 late-stage)	Advance care planning End-of-life needs assessments
7	Carlozzi et al.	2018 USA	Survey II	508 manifest or pre-manifest HD patients (114 late-stage)	Advance care planning End-of-life needs assessments
8	Carlozzi et al.	2019 USA	Survey II	507 manifest or pre-manifest HD patients (109 late stage)	End-of-life needs assessments
9	Dawson et al.	2004 Australia	Survey III	6 HD patients (1 middle/late stage); 19 informal carers (9 caring for HD patient in middle/late stage); 7 health care workers	Social care Health care services
10	Dellefield et al.	2011 USA	Narrative review V	n/a	Physical care Psychological care Social care Spiritual care Health care services
11	Downing et al.	2018 USA	Survey III	503 HD patients (113 late stage: TFC 0–6)	Advance care planning
12	Ekkel et al.	2021 the Netherlands	Survey III	12 patients (7 outpatient, 3 daycare, 2 assisted living facility)	Advance care planning
13	Hamedani et al.	2020 USA	Cross-sectional study IV	1614 hospital admissions for gastrostomy in HD patients	Physical care Health care services
14	Johnson et al.	2018 USA	Cross-sectional study IV	101 HD patients referred to hospice care, data extracted from CHOICE database	Physical care Social care Health care services
15	Kavanaugh et al.	2016 USA	Survey III	40 youth caregivers (children) of HD patients (age 12–20)	Social care Advance care planning
16	Kendrick et al.	2019 USA	Case study IV	1 pediatric HD patient from the age of 6 to 12	Social care Pediatric HD care Advance care planning
17	Kent	2015 UK	Narrative review V	n/a	Advance care planning
18	Kristjanson et al.	2006 Australia	Survey III	48 HD patients (4 receiving palliative care services); 56 HD caregivers (6 receiving palliative care services)	Physical care Health care services
19	Lamers	2006 USA	Case report V	1 HD patient receiving hospice care	Physical care Health care services
20	Lentz	2014 USA	Case report V	52-year-old HD patient, assisted-living facility	Health care services
21	Lindblad et al.	2010 Sweden	Survey III	667 physicians (internal medicine, surgery, intensive-care/anesthesiology, general practice and psychiatry); 625 general public	Advance care planning
22	Macleod	2005 New Zealand	Narrative review V	n/a	Advance care planning Health care services
23	Macleod et al.	2016 New Zealand	Narrative review V	n/a	Physical care Psychological care Social care Health care services
24	Marks et al.	2011 USA	Narrative review V	n/a	Physical care Psychological care Social care Advance care planning Health care services
25	Mendizabal et al.	2017 USA	Cross-sectional study IV	271 pediatric HD patients from the USA KID database, aged 5–20	Pediatric HD care
26	Mestre et al.	2017 Canada/USA	Narrative review V	n/a	Physical care Social care Advance care planning Health care services
27	Miller et al.	2012 USA	Case report V	13-year-old pediatric HD patient	Pediatric HD care
28	Moskowitz et al.	2001 USA	Narrative review V	n/a	Physical care Psychological care Advance care planning Health care services
29	Moskowitz et al.	2017 USA	Narrative review V	n/a	Physical care Psychological care Advance care planning Health care services
30	Phillips et al.	2008 UK/USA	Narrative review V	n/a	Physical care Psychological care Pediatric HD care Health care services
31	Roscoe et al.	2009 USA	Survey III	17 caregivers of late-stage HD patients (8 patients living at home, 7 in nursing home, 2 in assisted-living facilities or residential group homes)	Social care Spiritual care Health care services
32	Røthing et al.	2014 Norway	Survey III	15 family caregivers (aged 20–67) of HD patients (caring for parent, spouse, siblings or adult children)	Social care Health care services
33	Røthing et al.	2015 Norway	Survey III	15 family caregivers (aged 20–67) of HD patients (caring for parent, spouse, siblings or adult children)	Social care Spiritual care Health care services
34	Shah et al.	2021 Australia	Case report V	26-year-old patient referred to neuro-palliative ward	Advance care planning Health care services
35	Simpson	2007 UK	Narrative review V	n/a	Physical care Advance care planning Health care services
36	Sokol et al.	2021 USA	Survey III	322 patients (101 late stage)	Spiritual care
37	Sokol et al.	2021 USA	Cross-sectional study IV	8521 patients admitted to hospital, 321 receiving specialty palliative care	Health care services
38	Tarolli et al.	2017 USA	Narrative review V	5 HD patients (2 in advanced stage, 1 living in nursing home, 1 receiving hospice care)	Social care Advance care planning Health care services

HD: Huntington’s Disease

TFC: total functional capacity (mean, SD)

KID: Kids’ Inpatient Database

CHOICE: Coalition of Hospices Organized to Investigate Comparative Effectiveness

n/a: not applicable

**Table 2:**
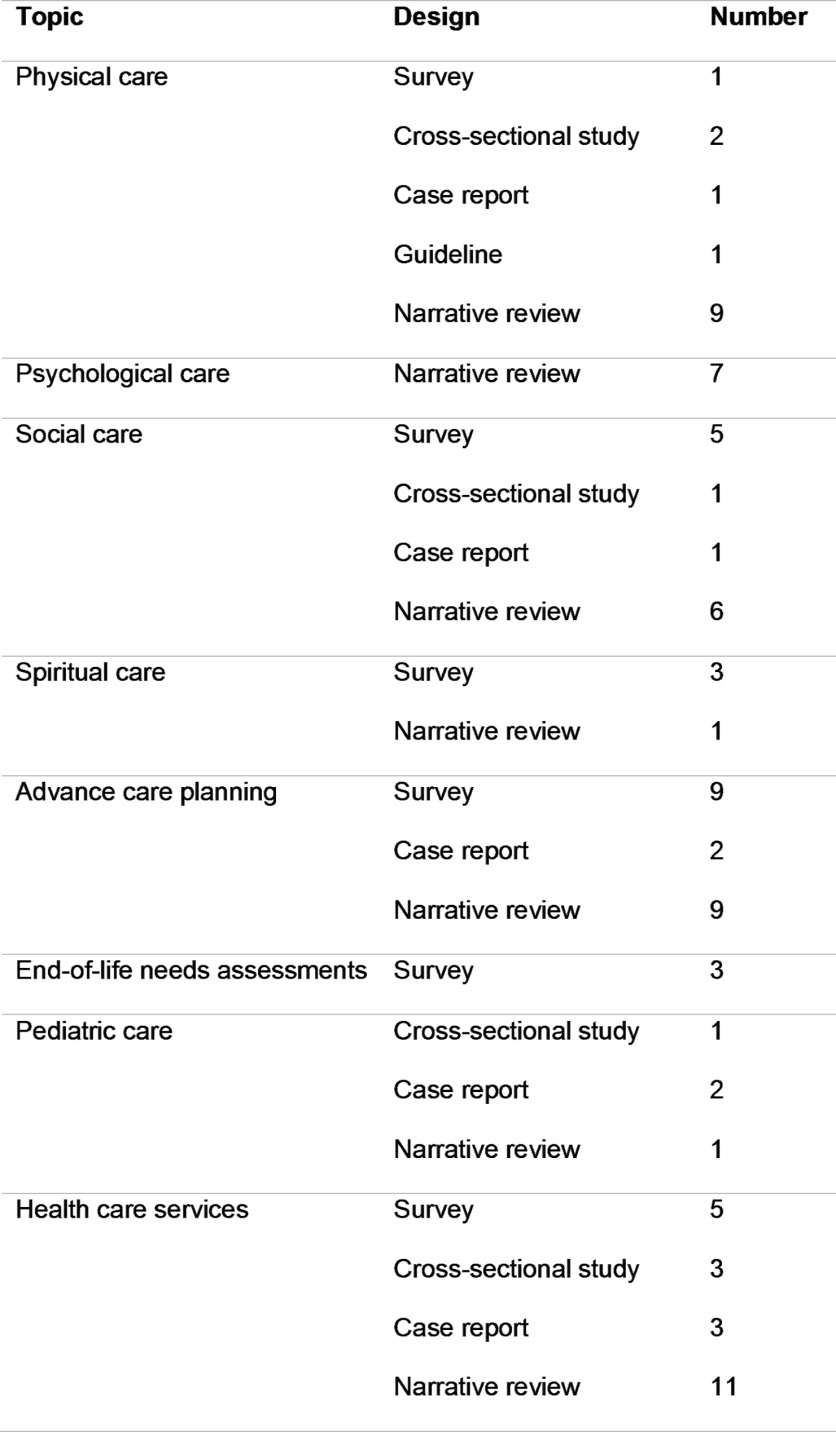
Study design sort by topic

### Care for physical symptoms

#### Motor symptoms

All eight included articles that describe the non-pharmacological management of motor symptoms are narrative reviews [9, 10, 21–26] based on expert opinion and experience. There are no systematic reviews or reports of clinical trials on non-pharmacological interventions in the advanced stage. Six articles describe options to prevent injuries by analyzing fall events, combined with gait and balance training and the use of orthotics [9, 10, 21–24]. Use of information technology may be useful to decrease the use of physical restraints [9, 10]. Independent functioning can be prolonged by functional adaptations such as padding of bed sides or sturdy furniture with rounded corners to prevent injury, wheelchairs with seat belts or padding, adaptations in utensils such as cutlery and tableware [9, 10, 21, 22, 24]. One article recommended muscle stretching to prevent contractures by stretching during nurse care and recreational activities, massages, or water therapy [9]. In case of muscular contractures, skin breakdown can be prevented by soft splints [9, 22, 26]. The use of photographs or objects can support communication [9, 22, 25] in case of progressive dysarthria [10, 21, 25].

#### Weight loss

Progressive weight loss in the advanced stage is described in five narrative reviews [9, 10, 21, 22, 24] and one guideline by dietitians from the European Huntington’s Disease Network (EHDN) which is based on consensus of the expert dietetic opinion because of lack of evidence [27]. Nutritional care may be challenging due to a variety of complicating symptoms, including obstipation, reduced mobility, dexterity and cognition, swallowing problems combined with food cramming, spillage or apathy [21, 22, 27]. High-calorie intakes are generally advised to prevent further weight loss [9, 10, 21, 22, 27].

#### Eating

Six narrative reviews [9, 10, 21, 22, 24, 25], one survey [28], and one guideline [27] referred to dysphagia. Patients with HD experience more swallowing problems compared to those with motor neuron disease, multiple sclerosis, and Parkinson’s disease [28]. A retrospective cross-sectional study described that 5% of HD hospital admissions in the USA include gastrostomy placement, which is mainly associated with aspiration pneumonia (34%), dementia (31%), malnutrition (30%) and dysphagia (29%) [29]. No clinical trials focusing on dysphagia have been conducted. To prevent aspiration pneumonia, swallowing therapy by a speech therapist is recommended [9, 10, 21, 27]. Strategies for safe swallowing are: attention for posture with leg weights and positioning to prevent regurgitation, avoiding distraction during eating, and providing assistance if needed [9, 21, 22, 24, 25, 27]. Thickened liquids and adapted consistency of food can prevent aspiration or choking [9, 21, 22, 24, 25]. Food with different textures in the mouth increases risk of choking and should therefore be avoided. Furthermore, it is recommended that nursing staff are able to apply the Heimlich maneuver in case of choking [21, 22]. Vomiting can be caused by food triggers, fear of choking or other problems causing volitional vomiting and incomplete mouth closure [9, 21]. PEG feeding is rarely used in advanced-stage patients [10, 22, 24]; there is an ethical debate about the use of PEG feeding [30, 31], which should be considered individually [9, 10, 21, 24, 25, 27].

#### Dental care

In five narrative reviews [9, 21, 22, 24, 27] the importance of dental care to control bacterial growth and decrease the risk of infections is emphasized. Poor oral health can also affect communication and may result in feeding complications. No clinical trials are available. Three articles present practical recommendations, for example using electric tooth brushes and peroxide for better bacterial control [21, 22], and attention for ill-fitting dentures as a result of cachexia [9].

#### Sleep

Three narrative reviews describe sleeping problems that are related to circadian rhythm disturbances [22, 24, 25]. A pre-bedtime routine decreases anxiety and promotes sleep. High-calorie snacks are recommended during the night for patients who wake up hungry [9]. Other adaptations such as a low double-bed with four padded sides can be helpful to lessen injury [9, 21].

#### Pain

Although chronic pain [13, 26], which may arise from hyperkinetic movements and injury, hypokinesis, dystonia or spasticity [10, 22, 24], is more common in the advanced stage than in earlier stages, no publications on pain management in the advanced stage were found.

#### Other

Smoking becomes increasingly hazardous when patients have severe motor or cognitive symptoms, and requires specific attention, although involuntary deprivation of smoking has ethical implications [22, 24]. Bladder and/or bowel incontinence can be eased by toileting regimes [22], and autonomic hypofunction as a result of neurodegeneration of autonomic centers [24] has been described, such as excessive perspiration, and disturbances of temperature regulation and blood pressure [9, 22, 24], which can be lethal [22]. Sensory-type programs can be beneficial for well-being and enjoyment in advanced stage HD [10].

### Psychological care

#### Challenging behavior

In the advanced stage, unexplained screaming, crying and agitation may occur. Irritability may escalate to violent behavior in some patients, whereas other patients become increasingly apathetic [9, 10, 25] or impulsive [10, 25]. We found four narrative reviews that describe behavioral management in the advanced stage. Daily routines are advised and may decrease behavioral disruptions [9, 10, 25] [22]. It is recommended to identify triggers and situations in which patients become angry or irritable to avoid these situations [10, 22, 25]. Training nursing staff in responding to violence or aggression with a de-escalating approach is advised [10].

#### Neurocognitive dysfunction

Neurocognitive impairments in the advanced stage include inflexible thinking, perseveration, severe bradyphrenia, disinhibition. These are discussed in seven narrative reviews [9, 10, 21–25]. In general, it is advised to be aware of patients’ unawareness of deficits [21]. Using short sentences and questions in communication and waiting for acknowledgment or response is advised [21, 23]. It is recommended to give verbal and visual cues to aid retrieval [21, 22, 25], to present no more than two choices instead of open-ended questions, to try to distract in case of perseveration, and give a positive response to repeated demands [9, 22, 25].

### Social care

Thirteen articles, including five surveys [32–36], one cross-sectional study [13], one case report [37], and six narrative reviews [10, 22, 23, 25, 26, 38], describe the importance of involving family caregivers in palliative care. Attention to family members during palliative care may improve the quality of life and reduce psychological distress [10, 13, 25, 38]. The risk of having inherited the HD gene and developing the disease in the future are stressors experienced by offspring in families with HD [23, 33, 34, 36]. The burden of the chronic, progressively neurodegenerative disorders may cause caregiver burnout and depression [22, 26, 33, 34, 38], and disturbed family relationships and social isolation in HD families are described [10, 25, 26, 32–35, 37, 38].

### Spiritual care

One survey showed that a sense of meaning and purpose is associated with increased positive affect and well-being, while psychiatric symptoms, like depression and anger, may improve [39]. These spiritual needs were more frequently met through consistent relationships between residents, family and staff, instead of religious counsellors [10]. A study which combined interviews with the 22-item Spiritual Involvement and Beliefs Scale-Revised, showed that providing care for loved ones is meaningful for caregivers and gives a positive experience. They also reported a sense of control over life problems, and could find meaning in times of hardship [33]. An interview study described that family caregivers generally accept the HD diagnosis, but were hesitant to talk to others about their situation and chose to be open about HD when they considered it appropriate [35]. Feelings of guilt, regret, and sadness because of passing the gene to family members are described by expert opinion [10].

### Advance care planning

Twenty articles addressed advance care planning (ACP) in surveys [36, 40–47], narrative reviews [9, 21, 23, 25, 26, 30, 38, 48, 49], and two case reports [37, 50]. Having close relatives with HD was significantly associated with the existence of end-of-life (EOL) wishes [30, 40–42, 44, 47, 48]. To promote patients’ autonomy, conversation about EOL wishes is considered important [23, 25, 26, 30, 36–38, 40, 41, 47]. EOL planning for patients with HD is advised to reduce emotional distress from unwanted EOL care [9, 21, 30, 46]. It may also be a source of comfort to relatives who can then be relieved of the burden of making life-or-death decisions for their relative of partner [21, 23, 30, 36, 48, 49]. Talking about retaining quality of life until the end of life may result in talking about quality of dying [40], and can be facilitated by neuro-palliative care services [50]. However, cognitive decline and psychiatric symptoms [21, 38, 40, 48, 49], or avoidance [47] may complicate discussing EOL wishes. In the HDQLIFE-study in the USA, 38.2% of participants had an advance directive [46]. Two Dutch studies about EOL wishes found that most EOL wishes concerned euthanasia and physician-assisted suicide and were more frequently discussed with family than with the patient’s physician [41, 42], which was confirmed by findings of Carlozzi et al. [45] in the USA. Another survey among physicians discussed that talking about EOL wishes is the legal, professional, and moral responsibility of the physician [40]. A Swedish survey among physicians and individuals about a vignette describing a HD patient who is refused physician-assisted suicide and is offered continuous deep sedation instead, suggesting that there is need for a broader discussion about the recommendations for continuous deep sedation [43].

### End-of-life needs assessments

Three articles describe HDQLIFE measures that evaluate concerns at the end of life in HD. The first publication describes the development of two measures: a 4-item scale for Meaning and Purpose, and a 6-item form to capture Concern with Death and Dying [44]. These measures may help to initiate conversations about wishes and EOL care, and can be used to identify distress in patients who might benefit from mental health services. The second publication reports about the development and testing of a 16-item End of Life Planning measure which showed adequate psychometric properties [45]. This measure includes 4 subscales: legal planning, preferences for care, preferences for death and dying, and financial planning. The purpose of this tool is to have productive and meaningful discussions about EOL planning. There is strong psychometric support for the reliability and validity of these three measures [51].

### Pediatric HD care

Two case reports [37, 52], one cross-sectional study [53], and a narrative review [24] discuss palliative care in pediatric HD patients (onset < 18 y/o) who have a different phenotype compared to adult HD patients, which is characterized by physical symptoms, including dysarthria, epilepsy, myoclonus, dystonia, spasticity, and ataxia, and psychological symptoms, such as early cognitive impairment, behavioral symptoms, hallucinations and delusions [24, 37, 52]. For family caregivers it is often difficult to find adequate respite care due to unfamiliarity with pediatric HD [37]. Reasons for referral of pediatric HD patients to hospitals are epilepsy, seizures and convulsions, followed by psychiatric diagnosis and respiratory infection/aspiration; the most common procedure was percutaneous endoscopic gastrostomy and endotracheal intubation [37, 53]. A case study described the value of extensive EOL conversations on hospice care, and of hospice care in avoiding hospital readmission [52], as children with pediatric HD were 8 times more likely to die during their hospitalization than other hospitalized children [53].

### Health care services

Twenty-two articles refer to health care services as significant for providing palliative care. Two narrative reviews [26, 30], and two surveys [32, 33] described that finding suitable inpatient care is often stressful for caregivers. As HD is less known than other neurodegenerative diseases, and HD patients, compared to other nursing home residents, are younger, more mobile and spend more years in nursing homes [9, 13, 28], institutions lack experience and may wish to avoid (deliberately taken) risks such as risk of aspiration, falls, going outside unaccompanied and behavioral issues, as described by two narrative reviews [10, 30], two surveys [32, 34], and two case reports [50, 54]. To increase safety and independence, and decrease anxiety and boredom, a specific environment is needed. Examples of adaptations are given in three narrative reviews [10, 21, 22] and include wide doorways, grab bars on the wall, open and well-lit spaces for unfettered views and rooms at a cool temperature. A multidisciplinary team is valuable as described by six narrative reviews [23–25, 30, 38, 49], a survey [32] and two case reports [50, 54].

Respite care can be used as preparation for the advanced stage [24, 30], also to reduce the burden placed on the (young) carer [21, 22, 32, 35, 38]. When a patient can be admitted to a nursing home or hospice, a neuro-palliative ward is preferred [50]. Reasons for institutionalization are disability or death of a caregiver, escalating needs that cannot be met at home, or after discharge from a hospital [9, 30]. Referral to palliative care of HD patients in the USA is associated with a higher mortality class, DNR status, aspiration pneumonia, and respiratory failure [55]. Considering the total hospice population, HD patients represent less than 1% [13], but indications and prediction of prognosis for hospice enrollment or referral to palliative care may be unclear in case of HD [13, 23, 31, 38].

Nursing home placement can be delayed by implementation of palliative care that focuses on community support [28, 29, 35], and families who prefer to care for relatives at home should be supported [24].

## Discussion

In this review eight topics of palliative care were identified: physical care, psychological care, social care, and spiritual care reflecting the definition of palliative care; and additional topics on advance care planning, EOL needs assessments, pediatric HD care, and health care services. The level of evidence for non-pharmacological management of physical and psychological symptoms is low (mainly Level V), and not very specific for the advanced stage of HD. However, patients often report problems with physical or functional issues and to a lesser extent psychological and emotional problems in later stages [56]. We found little reference to a more upstream orientation towards palliative care for HD, as even the dying phase is understudied. However, we found support for ACP conversations which contributes to this upstream orientation. We recommend that additionally symptoms and problems with increasing severity and progression of the disease up to the advanced stage HD be monitored, to tailor treatment and approaches accordingly.

Spiritual care is understudied in the advanced stage in HD. More attention is given to social care (evidence Level III-V) and advance care planning (evidence Level II-V). The other care-related topic of end-of-life needs assessments rated a Level II or III, whereas the evidence level of the topics on pediatric HD care and health care services was low.

The literature on physical and psychological care problems are predominantly focused on pharmacological management and rarely described an evidence-based non-pharmacological approach. Although pharmacological management of physical and psychological symptoms in the advanced stage were not included in this review, there is a lack of guidelines on medication use in the very last stage, especially the need for and use of psychopharmaceuticals [9, 21, 24, 25].

Dysphagia is a common problem in HD and often leads to aspiration and discomfort [57, 58]. It is unclear whether tube feeding prolongs survival time for HD [25], which also applies to Parkinson’s disease or related disorders [59]. Additionally, tube feeding may also lead to discomfort [60] and may not be applied because of ethical considerations [17, 29–31]. More research is needed to better understand the different perspectives in this debate.

There is little research on occurrence of pain in the advanced stage. For optimal treatment it is important to know the prevalence of pain and the best way to treat it to improve quality of life in the advanced stage and quality of dying in the terminal stage. HD patients may be unaware of pain, although the distress it may cause is also unknown. Sprenger et al. describe in a systematic review [61] that the burden of pain seems to be lower in HD patients, compared with the general population, but that it is comparable to those with other neurodegenerative disorders.

Social care and caregivers’ experiences are studied in a number of surveys [32–36, 56]. It is recommended to involve family in palliative care and ACP conversations [10, 13, 25, 38], as patients often already do [41, 42]. Patients appreciate the opportunity to discuss EOL-care, but may expect the physician to start the conversation, although this appears to be uncomfortable for many physicians [62, 63]. Patients often have clear ideas or wishes about their future, and are willing to talk about them, although Ekkel et al. found that patients tended to keep the future at a distance [47]. To start ACP conversations, Carlozzi et al. developed and tested three assessment scales [44, 45, 51]. They recommend the use of these tools to capture patients’ preferences about EOL, which include legal and financial planning and preferences related to care, death, and dying. We suggest to use these assessment tools which can also help to identify and to discuss spiritual needs at the end of life. Requests for ACP conversations may be mainly initiated by patients in an earlier stage compared to the advanced stage, but Booij et al. describe that most patients with cognitive deterioration are still able to speak about their wishes [41], including euthanasia if this allowed. ACP conversations may contribute to the upstream orientation. Additionally, ACP can help identify the palliative care needs in an earlier stage of HD, and to discuss patients’ preferences about the moment of introducing palliative care. A ‘good death’ is important for both patient and family, and most important is fulfilling wishes regarding the dying process [64], especially since witnessing a loved one in the final stages proves to be important for the EOL choices they will make later on themselves [30, 40–42, 44, 48]. Recommendations for deep sedation as an alternative to euthanasia should be discussed, as suggested in a survey [43], but in our opinion it is not preferred as the recommendation, according to most guidelines for continuous deep sedation, is to start only in the last days or weeks [65, 66], and the terminal stage is difficult to predict in HD [13, 23, 31, 38, 50].

Health care services are frequently needed in an advanced stage although a lack of knowledge about HD among health care providers has been reported [32, 38, 50, 56, 67]. Recognizing symptoms in the end-of-life stage is important for adequate and timely referral to palliative care (units) and to avoid unwanted hospitalizations. Indications for referral to palliative care are not clear [16], and are not studied in HD [13, 23, 31, 38, 50], which may lead to poor recognition of need for hospice benefit, also in children with pediatric HD where EOL conversations may guide improvement of quality of life and dying by avoiding hospitalization [52, 53]. In HD, the strongest predictor for nursing home admission is advanced motor impairment. Severe behavioral problems are common in nursing home HD patients, but in general do not predict admission [68]. Nursing home placement should preferably be in specialized HD care units, to avoid HD patients and their family feeling a lack of support from health care professionals in late stage. If this is not possible, staff should be informed about HD and trained to learn skills [10, 21, 22, 69] such as behavioral modification strategy [70].

None of the articles we found studied the very last stage, except regarding causes of death. Although the importance of striving for a good end of life is obvious, previous and ongoing research often focuses on pathophysiology and cure, and much less on care and improvement of quality of life [71–73]. This reflects a research gap and is in contrast to other neurodegenerative diseases such as Parkinson’s disease [17, 74] and dementia [75] which are similar in some ways. Van der Steen et al. conducted a review [76] that discusses similarities and differences between palliative care in Alzheimer’s disease and Parkinson’s disease. Similar to HD, the later stages are difficult to demarcate. However, the prediction of mortality is better studied for Alzheimer’s disease and Parkinson’s disease than for HD. Non-pharmacological approaches are also better studied in Parkinson’s disease and Alzheimer’s disease compared to HD. Assessment tools such as the ‘Integrated Palliative care Outcome Scale for Dementia’ assessment tool for dementia and the ‘Palliative care Outcome Scale Parkinson disease’ are available [76]. These are useful to observe multiple symptoms and palliative care needs respectively, and may be also useful for HD patients.

Some problems in the last stage, such as withdrawal of medication due to swallowing problems, leading to discomfort, or the management of refractory agitation warrant further research. Identification of the symptoms and characteristics predicting and demarcating the very last stage also needs further research, since they can contribute to quality of life and dying [17]. Also, there is a lack of knowledge of the needs and wishes of caregivers around the patient in the terminal stage.

### Strength and limitations

The strengths of this scoping review are the broad literature search focusing on the advanced stage of HD, which shows the state of research activity on palliative care in HD. Second, the literature is divided into eight topics, reflecting the definition of palliative care and other care-related topics describing specific characteristics for HD. Limitations are the generally low level of evidence of the studies. Second, the wide range of study designs may also represent earlier stages of the disease or discuss a more general population as they have not always defined the stages clearly. Third, we did not search ‘grey literature’, that may include helpful practical advises for palliative care.

## Conclusion

In the literature, palliative care focuses on physical problems and to a lesser extent on social care and advance care planning. Further, understudied topics include psychological and spiritual care, health care service use, EOL needs assessments, and care for pediatric patients. It is unclear to what extent palliative care for HD is provided in practice and what an effective palliative intervention entails. Further research is needed on implementation and evaluation of palliative models of care for persons with HD and their family caregivers.

## Electronic supplementary material

Below is the link to the electronic supplementary material.

**Supplementary Material 1: Table 1.** Full search strategy for each database.

## Data Availability

See Supplementary Table 1 for the full search strategy for each database.
