# Correction to: palliative care in advanced Huntington’s disease: a scoping review

**DOI:** 10.1186/s12904-023-01186-5

**Published:** 2023-05-31

**Authors:** Dorine J. Boersema-Wijma, Erik van Duijn, Anne-Wil Heemskerk, Jenny T. van der Steen, Wilco P. Achterberg

**Affiliations:** 1 Department of Public Health and Primary care, Leiden University Medical Center, Hippocratespad 21, Leiden, 2333 ZD the Netherlands; 2 Huntington Center of Expertise Topaz Overduin, Nachtegaallaan 5, Katwijk, 2225 SX the Netherlands; 3 Radboudumc Alzheimer center and Department of Primary and Community Care, Radboud university medical center, Geert Grooteplein, Noord 21, Nijmegen, 6500 HB the Netherlands

**Correction: BMC Palliat Care 22, 54 (2023).**

10.1186/s12904-023-01171-y.

Following publication of the original article [1], the author reported that some requested changes in the affiliation during corrections stage were not implemented.

- Erik van Duijn and Wilco Achterberg affiliations should be 1 and 2.
- Format of affiliations at the end should be: Street + number – Zip code – City – Country.

The original article has been updated.
