# Supplementary material for: Palliative care in advanced Huntington’s disease: a scoping review
Source: BMC Palliat Care. 2023 May 3;22:54. doi: 10.1186/s12904-023-01171-y (PMC10155365; doi:10.1186/s12904-023-01171-y)
Supplement: Supplementary file 1 — Supplementary Material 1: Table 1. Full search strategy for each database. [file 12904_2023_1171_MOESM1_ESM.docx]

Supplementary Table 1. Full search strategy for each database.

| Database | Number of articles | Search terms |
| --- | --- | --- |
| Academic Search Premier | 23 | TI(("Huntington Disease" OR "Huntington Chorea" OR "Huntington Disease" OR "Huntington*") AND ("Palliative Therapy" OR "Palliative Nursing" OR "terminal care" OR "terminal care" OR "Palliat*" OR "hospice care" OR "hospice" OR "hospice patient" OR "hospice nursing" OR "Hospice*")) OR SU(("Huntington Disease" OR "Huntington Chorea" OR "Huntington Disease" OR "Huntington*") AND ("Palliative Therapy" OR "Palliative Nursing" OR "terminal care" OR "terminal care" OR "Palliat*" OR "hospice care" OR "hospice" OR "hospice patient" OR "hospice nursing" OR "Hospice*")) OR KW(("Huntington Disease" OR "Huntington Chorea" OR "Huntington Disease" OR "Huntington*") AND ("Palliative Therapy" OR "Palliative Nursing" OR "terminal care" OR "terminal care" OR "Palliat*" OR "hospice care" OR "hospice" OR "hospice patient" OR "hospice nursing" OR "Hospice*")) OR AB(("Huntington Disease" OR "Huntington Chorea" OR "Huntington Disease" OR "Huntington*") AND ("Palliative Therapy" OR "Palliative Nursing" OR "terminal care" OR "terminal care" OR "Palliat*" OR "hospice care" OR "hospice" OR "hospice patient" OR "hospice nursing" OR "Hospice*")) |
| Cochrane Library | 0 | (("Huntington Disease" OR "Huntington Chorea" OR "Huntington Disease" OR "Huntington*") AND ("Palliative Therapy" OR "Palliative Nursing" OR "terminal care" OR "terminal care" OR "Palliat*" OR "hospice care" OR "hospice" OR "hospice patient" OR "hospice nursing" OR "Hospice*")):ti,ab,kw |
| Embase | 16 | ((*"Huntington Disease"/ OR *"Huntington Chorea"/ OR "Huntington Disease".ti,ab OR "Huntington*".ti,ab) AND (*"Palliative Therapy"/ OR *"Palliative Nursing"/ OR exp *"terminal care"/ OR "terminal care".ti,ab OR "Palliat*".ti,ab OR *"hospice care"/ OR *"hospice"/ OR *"hospice patient"/ OR *"hospice nursing"/ OR "Hospice*".ti,ab)) |
| Emcare | 10 | ((*"Huntington Disease"/ OR "Huntington Chorea"/ OR "Huntington Disease".ti,ab OR "Huntington*".ti,ab) AND (*"Palliative Therapy"/ OR *"Palliative Nursing"/ OR exp *"terminal care"/ OR "terminal care".ti,ab OR "Palliat*".ti,ab OR *"hospice care"/ OR *"hospice"/ OR *"hospice patient"/ OR *"hospice nursing"/ OR "Hospice*".ti,ab)) |
| PMC Pubmed Central | 99 | (("Huntington Disease"[Text Word] OR "Huntington Chorea"[Text Word] OR "Huntington Disease"[Text Word] OR "Huntington*"[text word]) AND ("Palliative Therapy"[Text Word] OR "Palliative Nursing"[Text Word] OR "terminal care"[Text Word] OR "terminal care"[Text Word] OR "Palliat*"[Text Word] OR "hospice care"[Text Word] OR "hospice"[Text Word] OR "hospice patient"[Text Word] OR "hospice nursing"[Text Word] OR "Hospice*"[text word])) |
| PsycINFO | 74 | TX(("Huntington Disease" OR "Huntington Chorea" OR "Huntington Disease" OR "Huntington*") AND ("Palliative Therapy" OR "Palliative Nursing" OR "terminal care" OR "terminal care" OR "Palliat*" OR "hospice care" OR "hospice" OR "hospice patient" OR "hospice nursing" OR "Hospice*")) |
| PubMed | 95 | (("Huntington Disease"[Mesh] OR "Huntington Disease"[tw] OR "Huntington*"[tw]) AND ("Palliative Care"[Mesh] OR "Hospice and Palliative Care Nursing"[Mesh] OR "Palliative Medicine"[Mesh] OR "terminal care"[mesh] OR "terminal care"[tw] OR "Palliat*"[tw] OR "Hospice*"[tw])) |
| Web of Science | 26 | TS=(("Huntington Disease" OR "Huntington Chorea" OR "Huntington Disease" OR "Huntington*") AND ("Palliative Therapy" OR "Palliative Nursing" OR "terminal care" OR "terminal care" OR "Palliat*" OR "hospice care" OR "hospice" OR "hospice patient" OR "hospice nursing" OR "Hospice*")) |
